# Using Genomic Data to Infer Evolutionary Processes in the Asexual Parasitoid *Microctonus aethiopoides*

**DOI:** 10.1002/ece3.72533

**Published:** 2025-12-26

**Authors:** Meeran Hussain, Elahe Parvizi, Mark R. McNeill, Ang McGaughran

**Affiliations:** ^1^ Te Aka Mātuatua ‐ School of Science University of Waikato Hamilton New Zealand; ^2^ Bioeconomy Science Institute Tuhiraki, Christchurch New Zealand

**Keywords:** arrhenotokous, biocontrol success, classical biological control, genetic variability, population genomics, thelytokous

## Abstract

Biological control offers a sustainable alternative to pesticides, with asexual parasitoids favoured for their ability to reproduce without males and produce all‐female offspring. However, asexuality may limit the parasitoid's long‐term adaptability to parameters, such as climate changes or physiological or behavioural changes in the host, reducing the potential effectiveness of the biocontrol. In this study, we performed whole‐genome resequencing on 43 individuals of the Irish strain of the asexual (thelytokous) endoparasitoid *Microctonus aethiopoides*, which was introduced to New Zealand in 2006 to control clover root weevil (*Sitona obsoletus*). We sampled from five parasitoid populations, one from the original collection location in Ireland and four release locations in New Zealand, to assess spatial and temporal genetic variation and investigate the genetic basis for its reproductive mechanism. Population structure analyses revealed two distinct genetic clusters, likely reflecting the differential establishment of haplotypes introduced from Ireland rather than geographic isolation. Though one haplotype appeared more widespread, particularly in the South Island, further sampling is needed to confirm this. All populations exhibited uniformly low genetic diversity, with Tajima's *D* values decreased in contemporary populations, indicating population expansion since introduction. Patterns of high heterozygosity and short homozygous segments support automictic thelytoky, likely via central fusion, as the primary mode of asexual reproduction. However, linkage disequilibrium rates resembled those of sexual populations, suggesting possible facultative sexual reproduction in this species. Overall, our genomic data provide new insights into how asexual biocontrol agents persist and evolve post‐release and shed light on this species' long‐term viability and reproductive strategy.

## Graphical abstract

In this study, we used whole‐genome resequencing to investigate spatial and temporal genomic variation in the Irish strain of the asexual (thelytokous) parasitoid *Microctonus aethiopoides*, which was introduced to New Zealand in 2006 to control clover root weevil (*Sitona obsoletus*). Our dataset comprised 43 individuals sampled from five populations, including the original Irish source and four New Zealand regions. We uncovered two major genetic clusters, likely reflecting differential establishment of introduced haplotypes rather than geographic isolation. Genomic signatures, including high heterozygosity and short homozygous tracts, support automictic thelytoky via central fusion as the primary mode of reproduction, while unexpectedly low linkage disequilibrium suggests possible facultative sexual reproduction.

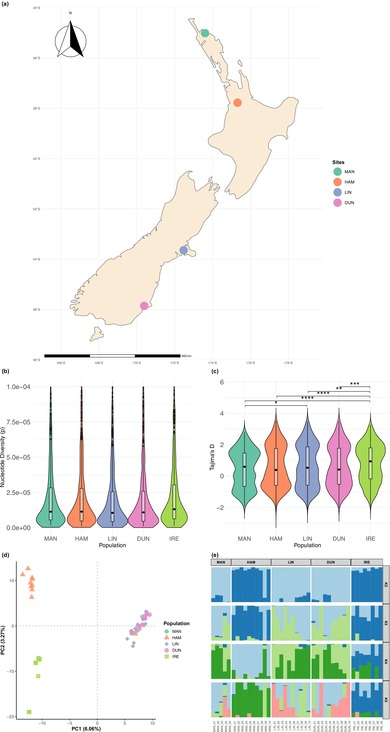

## 1 Introduction

Classical biological control is a key strategy for managing invasive species by introducing natural enemies from their native range. In New Zealand, exotic and accidentally introduced weevils, such as *Listronotus bonariensis* (Kuschel) (Argentine stem weevil) and *Sitona obsoletus* (Gmelin) (clover root weevil), are among the most serious pests of ryegrass (*Lolium* L. spp.) and white clover ( *Trifolium repens* L.), respectively. These pests have been estimated to cause up to NZ$235 million per year in damage to dairy, sheep, and beef pastures (Ferguson et al. 2019). Parasitoids are particularly valuable biocontrol agents in agriculture (Seehausen et al. 2021), and classical biological control programmes targeting both *L. bonariensis* (Barker and Addison 2006; Goldson et al. 1998) and *S. obsoletus* (Gerard et al. 2011; Goldson et al. 2005) have played an important role in their management. However, not all classical biocontrol efforts are effective.

Various factors can impact biocontrol success, including climate incompatibilities, mismatches between parasitoid strains and target hosts, physiological defences of the host, Allee effects and the influence of endosymbionts (Cameron et al. 1993; Hoelmer and Kirk 2005; Seehausen et al. 2021). An interesting example of apparent failure after initial success was the biocontrol of *L. bonariensis* by *Microctonus hyperodae* (Loan) (Hymenoptera: Braconidae). Although initially successful (Barker and Addison 2006; Goldson et al. 1998), levels of parasitism showed a progressive decline 7 years after initiation (Goldson et al. 2014), with a key hypothesis being that the asexual parasitoid was being outcompeted by its sexual host because of avoidance behavioural changes (Casanovas et al. 2018).

Being asexual clearly has certain advantages in terms of negating the need to find a mate under stable environments (Amat et al. 2017), and allowing for rapid population growth (Heimpel and de Boer 2008). Indeed, some 500 Hymenopteran species have been identified as having thelytokous asexual reproduction (van der Kooi et al. 2017). However, how asexual parasitoids maintain their effectiveness in dynamic and competitive ecological environments is relatively unexplored. Considering the apparent reduced effectiveness of *M. hyperodae* (Casanovas et al. 2018), this sets up an intriguing hypothesis about the role of reproductive mode in driving success/failure outcomes of classical biological control programmes.

A key limitation of asexuality is the potential associated reduction in genetic diversity. In the absence of a male partner, the lack of allelic reshuffling through meiosis between two parents results in offspring that are genetically similar to their mother, potentially limiting the population's overall ability to adapt to changing environmental conditions or evolving host defenses (Normark et al. 2003; Tvedte et al. 2019). In contrast, sexual reproduction introduces genetic recombination through meiosis, which enhances genetic diversity and provides greater potential for adaptation to environmental changes and host resistance (Heimpel and de Boer 2008; Otto and Lenormand 2002).

Yet, asexual reproduction is not universally clonal. Some asexual systems possess mechanisms that can preserve or generate genetic variation. For instance, in apomictic (strictly clonal) asexuals, new genetic variation arises only through the accumulation of spontaneous mutations, which is typically a slow process. However, in automictic asexuals, reproduction involves partial meiosis followed by the fusion of meiotic products from the same individual. Depending on the mechanism (central fusion, terminal fusion or gamete duplication), automixis can retain varying degrees of heterozygosity, thereby maintaining some genetic diversity despite the absence of fertilisation (Card et al. 2021; Lampert 2008; Pearcy et al. 2011).

Patterns of linkage disequilibrium (LD) can be used to provide insights into reproductive mode, as LD tends to break down in sexually reproducing populations due to recombination, whereas in asexual or inbred populations, LD remains high—reflecting reduced genetic reshuffling and the accumulation of linked alleles, a consequence similar to that observed under strong inbreeding (Balloux et al. 2003; Hartfield et al. 2018). Another strategy is facultative sexuality, in which organisms predominantly reproduce asexually but switch to sexual reproduction under certain conditions. This dual mode allows occasional gene flow when the asexual population co‐exists with its sexual conspecifics, enabling the retention or reintroduction of genetic diversity when needed (Adachi‐hagimori et al. 2011; Sandrock et al. 2011; Schneider et al. 2003). This diversity of asexual mechanisms highlights that even in the absence of regular outcrossing, some asexual organisms can mitigate the genetic limitations typically associated with clonal reproduction.

*Microctonus aethiopoides* (Hymenoptera: Braconidae) is an important endoparasitoid attacking the adult stage of *Hypera* and *Sitona* weevils (Aeschlimann 1983; Loan 1975; Loan and Holdaway 1961; Shaw 1988). Some strains have been successfully used in classical biological control programmes against agricultural pests in North America (Loan 1969; Radcliffe and Flanders 1998; Rand et al. 2018). In New Zealand, two strains of *M. aethiopoides* have been introduced to control two *Sitona* species. The Moroccan strain was introduced to New Zealand in 1982 to control *Sitona discoideus* (Gyllenhal) (lucerne weevil), a major pest of lucerne ( *Medicago sativa* L.) (Stufkens et al. 1987). Meanwhile, *Sitona obsoletus* (clover root weevil) was first detected in New Zealand in 1996 (Barratt et al. 1996). By 2006, it had rapidly spread to all parts of the North Island (Gerard et al. 2010), and it had reached most of the South Island by 2012 (Ferguson et al. 2012). Quarantine laboratory‐based studies showed that the Moroccan biotype of *M. aethiopoides* was ineffective against *S. obsoletus* (Barratt et al. 1997; McNeill et al. 2000), and this was attributed to a host physiological response mediated by endosymbionts that encapsulated and killed the developing eggs and larvae (White et al. 2015). Following extensive evaluation in quarantine (Goldson et al. 2005), an Irish strain of *M. aethiopoides* was thus released in 2006 (Gerard et al. 2006, 2007), and, within a year, parasitism rates of up to 70% were recorded in overwintering *S. obsoletus* populations (Gerard et al. 2011). There are distinct differences in reproductive mode between the two parasitoid biotypes. While Moroccan *M. aethiopoides* is a sexually reproducing (arrhenotokous) solitary endoparasitoid, the Irish strain is an asexually reproducing (thelytokous) and gregarious endoparasitoid (McNeill and Baird 2010). Although biocontrol of *S. obsoletus* is currently effective, the parasitoid's asexual reproductive mode and the sexual mode of its host suggest that degradation in the effectiveness of biocontrol may be a potential concern.

Here, we used genomic data to evaluate population diversity and differentiation patterns and explore reproductive mechanisms in the *M. aethiopoides* strain released against *S. obsoletus* . First, we assessed within and between population variation across space and time, using key population genomic metrics to assess the extent of genetic changes since the initial New Zealand releases in 2006. Second, we investigated patterns of LD and heterozygosity as informative metrics to infer whether recombination rates are suggestive of different forms of asexual reproduction in *M. aethiopoides*. Collectively, our approach provides a comprehensive understanding of how genomic factors may influence the long‐term effectiveness of this parasitoid as a biocontrol agent.

## 2 Materials and Methods

### 2.1 Sample Collection

Historic samples of *M. aethiopoides* (the Irish strain) that were preserved in 95% ethanol and stored at −80°C at the Bioeconomy Science Institute, Tuhiraki facility were obtained from collections made during quarantine‐based research conducted between 2000 and 2006 in England and New Zealand (Goldson et al. 2004; McNeill et al. 2006). Contemporary samples were reared from *S. obsoletus* collected from pastures containing white clover ( *T. repens* ) across four regions of New Zealand. In both Ireland and New Zealand, a modified blower‐vac (G‐vac) was used to collect litter from ryegrass‐white clover pasture (McNeill and van Koten 2020).

**FIGURE 1:**
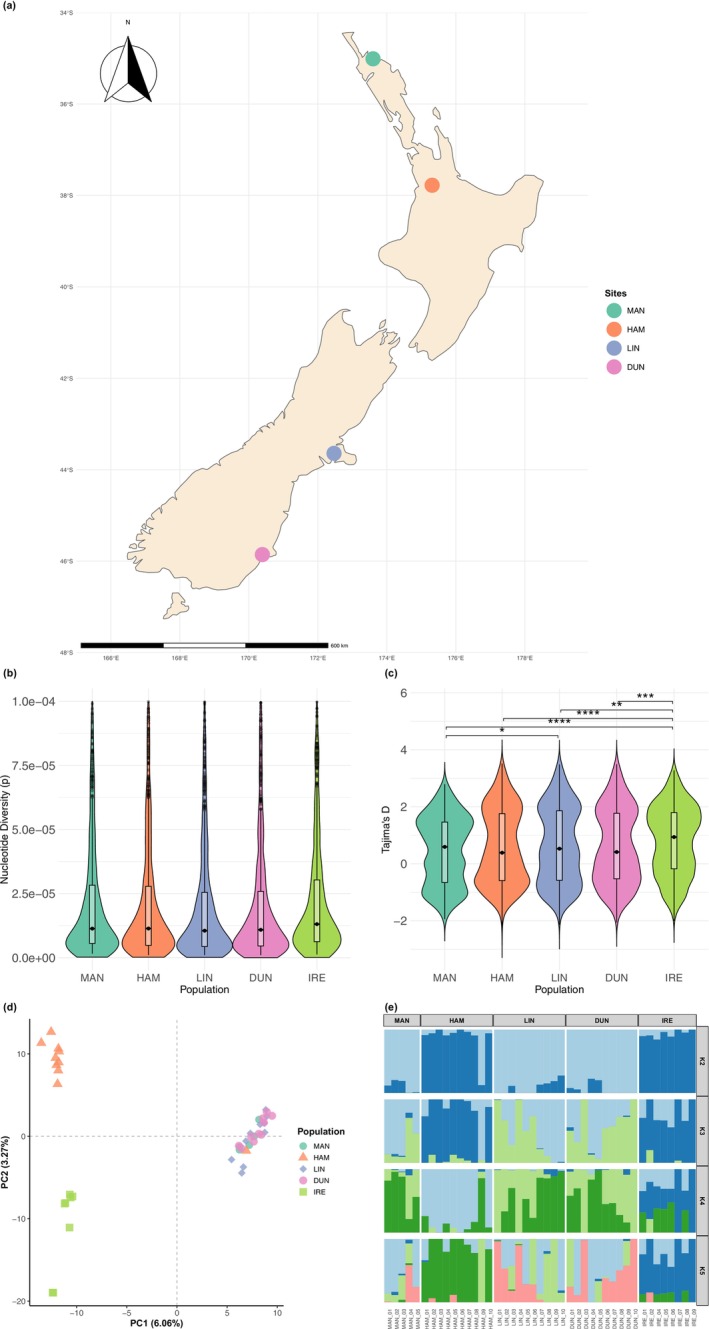
Sampling sites, genetic diversity and population structure of *Microctonus aethiopoides* (Irish) populations in New Zealand: (a) Map of New Zealand showing contemporary sampling sites used in this study: Mangōnui (MAN), Hamilton (HAM), Lincoln (LIN) and Dunedin (DUN). The base map was generated using Natural Earth data in R; (b) Violin plots of nucleotide diversity (π) per 100 kb window across five populations and (c) Violin plots of Tajima's *D* values per 100 kb window across five populations. Pairwise comparisons across populations were tested using the Wilcoxon rank‐sum test with Bonferroni adjusted *p*‐values, significance levels are indicated as: *p < 0.05, **p < 0.01, ***p < 0.001, ****p < 0.0001; comparisons without asterisks are not statistically significant (*p* ≥ 0.05); (d) Principal Component Analysis (PCA) showing genetic clustering of individuals from five populations; (e) ADMIXTURE analysis results for *K* = 2 to *K* = 5 ancestral clusters. Each vertical bar represents an individual, with colours indicating the proportion of ancestry assigned to each cluster. Individuals are grouped by population.

In the laboratory, *S. obsoletus* adults were separated from the litter, and parasitoids subsequently reared from weevils following established protocols described by McNeill et al. (2006). A total of 44 parasitoids were included in this study, comprising nine historic samples from Athenry, Ireland (IRE) and contemporary samples from Mangōnui (MAN, *n* = 5), Hamilton (HAM, *n* = 10), Lincoln (LIN, *n* = 10), and Dunedin (DUN, *n* = 10) (Figure 1a; Table S1). Contemporary parasitoid samples were preserved in 95% ethanol and stored at −20°C until DNA extraction.

### 2.2 DNA Extraction and Sequencing

Prior to DNA extraction, parasitoid samples were washed in deionised distilled water. Total genomic DNA was extracted from homogenised parasitoid samples using a column‐based proteinase K digestion method with the DNeasy Blood and Tissue Kit (Qiagen) according to the manufacturer's protocol. Extracted DNA was quantified using the Qubit dsDNA High Sensitivity (HS) Assay Kit (Invitrogen) and a Qubit Fluorometer, yielding DNA concentrations ranging from 113 to 1320 ng (Table S1). Illumina sequencing was performed to achieve approximately 30‐fold genome coverage. Paired‐end sequencing (2 × 150 bp) was conducted using PCR‐Free library preparation on a NovaSeq 6000 by Livestock Improvement Corporation (LIC) Genomics Facility (Hamilton, New Zealand).

### 2.3 Data Analysis

A high‐quality improved genome assembly of the Irish ecotype of *M. aethiopoides*, developed using Oxford Nanopore Technologies and Illumina sequencing for ongoing comparative genomic analysis (Hussain M, Parvizi E, McNeill M, McGaughran A; unpublished), was used in this study. The genome (131.1 Mb, comprising 231 scaffolds with 8 pseudo‐chromosomes) was assembled via ragtag scaffolding based on a previously Hi‐C scaffolded genome assembly (Inwood et al. 2023) and consists of 13,881 predicted genes with a BUSCO completeness score of 95.0%.

Of the 44 samples processed, 43 were successfully sequenced, while one sample from the Irish population (IRE) failed to yield usable data (Table S1). Raw Illumina reads from the remaining samples were quality‐filtered using Trim Galore (v0.6.10) (https://github.com/FelixKrueger/TrimGalore) with a minimum quality score (−q) of 20 and a minimum read length (−length) of 100 bp. Clean reads were aligned against the assembled reference genome using BWA‐MEM (v0.7.17) with M and R flags to mark secondary reads and add read groups, respectively (Li 2013). The resulting SAM files were processed with SAMtools (v1.16.1) to remove duplicate reads (markdup) and sort alignments (Danecek et al. 2021). Only uniquely mapped reads (‐F 4) were retained for variant calling. SNPs were called using BCFtools (v1.19) with a minimum mapping quality (‐‐min‐MQ) and base quality (‐‐min‐BQ) of 20 (Danecek et al. 2021). Biallelic SNPs were filtered to retain variants with a minimum minor allele frequency (MAF) > 0.05. Further filtering was performed on the resulting VCF file using PLINK v1.9 to retain SNPs with at least a 90% genotyping rate (‐‐geno 0.1) (Purcell et al. 2007). Additionally, individuals with more than 90% missing data (‐‐missing‐indv) were excluded using VCFtools (v0.1.15) (Danecek et al. 2011). These processing steps were integrated into a Snakemake pipeline, publicly available at (https://github.com/meeranhussain/Population_genomic_analysis). A total of 133,329 high‐quality SNPs were identified following these filtering steps (Table S2).

The population structure of *M. aethiopoides* was inferred using principal component analysis (PCA), implemented with the R package adegenet (v2.1.1) (Jombart and Ahmed 2011), and sparse non‐negative matrix factorisation (sNMF) analysis, performed with the R package LEA (v2.8.0) (Frichot and François 2015). Prior to these analyses, variants underwent a stringent filtering step to reduce LD using PLINK v1.9. Specifically, SNPs were filtered based on pairwise LD (‐‐indep‐pairwise) with a window size of 50 SNPs, a step size of 5 SNPs, and an *r* ^2^ threshold of 0.2, resulting in a total of 37,285 SNPs retained for population structure analyses. The sNMF analysis was conducted by evaluating up to eight putative clusters (*K*‐values ranging from 1 to 8), with 50 iterations of the algorithm performed per *K*‐value. Cross‐entropy results were plotted for each *K*‐value to determine the optimal number of genetic clusters.

Tajima's *D*, nucleotide diversity (π), and individual‐level heterozygosity were calculated across the genome in 100 kb sliding windows using VCFtools based on the full dataset of 133,329 high‐quality SNPs. Heterozygosity for each individual was estimated using the ‐‐het option and observed heterozygosity (Obs_Het) was calculated using the formula: Obs_Het = 1 ‐ (O(HOM)/N_SITES), where O(HOM) is the number of observed homozygous sites and N_SITES is the total number of sites analysed. Genetic differentiation (*F*_ST_) was assessed using pixy (v1.2.11. beta1) (Korunes and Samuk 2021). First, an all‐site VCF file was prepared to include both variant and invariant sites, following the official pixy documentation (pixy.readthedocs.io/en/latest/generating_invar/generating_invar.html). Weir and Cockerham's *F*_ST_ was then calculated using a sliding window size of 100 kb and results were plotted to visualise the outlier windows for each population.

To infer reproductive mode information from genetic data, LD decay was calculated using the full filtered dataset (133,329 SNPs). To account for uneven sampling among populations, five individuals were randomly selected from each population for this analysis. LD decay was calculated for all SNPs within 500 kb windows using the OutStat command implemented in PopLDdecay (v.3.31) (Zhang et al. 2019) and decay curves were plotted up to 10 kb to allow better resolution of the decay trend. Runs of homozygosity (ROH) were called across the full genome using PLINK v1.9 on a less filtered dataset without MAF filtering (to retain information on rare alleles) while applying a genotype missingness filter (‐‐geno 0.2), resulting in 160,071 SNPs. ROH segments were called using the ‐‐homozyg function with the following parameters: ‐‐homozyg‐kb 20, ‐‐homozyg‐snp 50, ‐‐homozyg‐window‐het 1, ‐‐homozyg‐density 40, ‐‐homozyg‐gap 100, and ‐‐allow‐extra‐chr. Using the results, *F*_ROH_ (a measure of inbreeding that quantifies the proportion of the genome made up of homozygous segments) was calculated as the total length of ROHs per individual divided by the total genome size (~130 Mb). Additionally, a phylogenetic tree was constructed to examine the genetic relationships among *M. aethiopoides* individuals/populations. The VCF file was converted to PHYLIP format using vcf2phylip (v.2.8) (Ortiz 2019), and a maximum‐likelihood tree was inferred with IQ‐TREE (v.2.2.2) (Minh et al. 2020) using 1000 ultrafast bootstraps (−bb 1000). The resulting tree was visualised and annotated using iTOL (v.6) (Letunic and Bork 2024). To evaluate the statistical significance of differences (e.g., Tajima's *D*, genetic diversity and ROH values) between populations, we used Wilcoxon rank‐sum tests with Bonferroni correction for multiple comparisons.

## 3 Results

### 3.1 Population Diversity and Differentiation

Nucleotide diversity (π) across the sampled populations of *M. aethiopoides* was consistently low, with median values ranging from 2.70e^−05^ to 3.24^e‐05^ and similar levels of genetic variation across both contemporary and historic samples (*p* > 0.05 in all Wilcoxon pairwise comparisons; Figure 1b). While Tajima's *D* median values were close to zero across all populations, we observed a significant decrease in values for the New Zealand contemporary versus the historic Irish (IRE) populations, indicative of population expansion (Figure 1c).

The population structure analysis indicated that *M. aethiopoides* in New Zealand most likely comprises two distinct lineages (Figure 1d). PCA showed clear clustering of the Hamilton (HAM) and Irish (IRE) populations along the first principal component (PC1), which explained 6.06% of the genetic variance. Individuals from Mangonui (MAN), Lincoln (LIN) and Dunedin (DUN) grouped closely in a second cluster, indicating minimal genetic differentiation among these contemporary populations. PC2, accounting for an additional 3.27% of the total variance, further differentiated the HAM and IRE populations.

The sNMF analysis supported the PCA results, with population admixture plots generated for *K* = 2–5. Although the cross‐entropy analysis did not show a clear optimal value (Figure S1), *K* = 2 was used for interpretation following the recommendation of Janes et al. (2017). At *K* = 2, the HAM and IRE populations were clearly distinguished from the remaining populations, with individuals in MAN, LIN and DUN showing up to 90% genomic apportioning to the light blue cluster (Figure 1e). Increasing the number of clusters (*K* = 3 to 5) showed finer‐scale differentiation within contemporary populations; however, overall patterns consistently highlighted the genetic uniqueness of the HAM and IRE populations compared to the other contemporary populations.

Pairwise *F*_ST_ values indicated moderate genetic differentiation between populations, with values ranging from 0.009 to 0.139 (Table 1). Consistent with PCA and ADMIXTURE results, the lowest differentiation was observed between MAN and LIN (*F*_ST_ = 0.009) and the highest between HAM and DUN (*F*_ST_ = 0.139). The Irish population (IRE) showed moderate differentiation from the contemporary New Zealand populations (*F*_ST_ = 0.106 to 0.127), suggesting the occurrence of some genetic divergence post‐introduction.

**TABLE 1:** Pairwise genetic differentiation (mean Weir and Cockerham's F_ST_) among *Microctonus aethiopoides* populations, calculated using variant and invariant sites across five populations.

	DUN	HAM	IRE	LIN
HAM	0.139			
IRE	0.127	0.106		
LIN	0.009	0.137	0.124	
MAN	0.014	0.135	0.117	0.012

Genome‐wide *F*_ST_ analysis revealed that outlier loci were widely dispersed across the genome and were primarily observed in population comparisons that involved HAM and IRE (Figures S2 and S3). However, the observed pattern showed no clear evidence of increased differentiation in any specific genomic region.

### 3.2 Genomic Patterns of Linkage and Recombination

**FIGURE 2:**
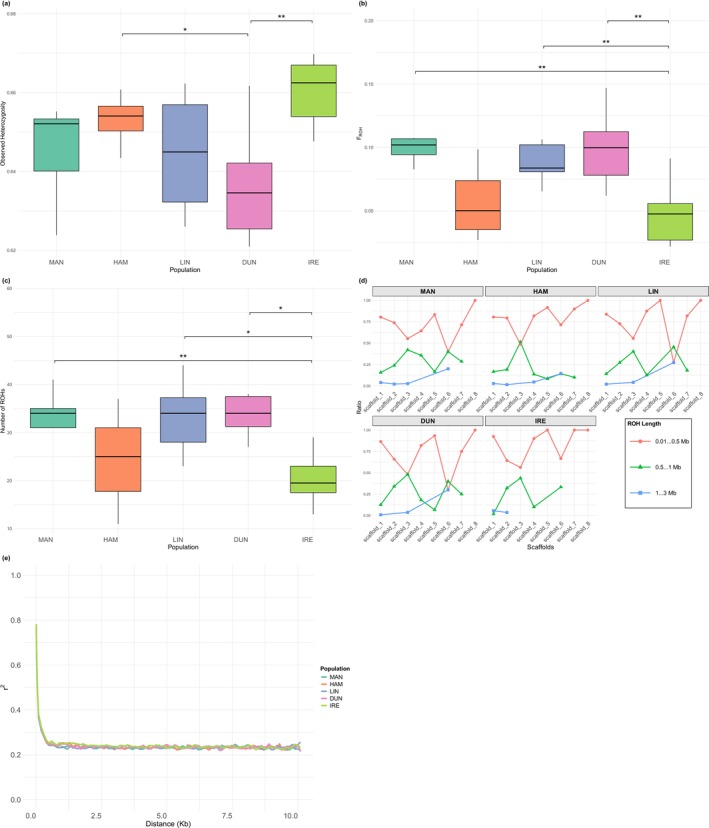
Heterozygosity, homozygosity, and linkage estimates across *Microctonus aethiopoides* populations: (a) Observed heterozygosity per individual; (b) Genomic inbreeding coefficients (*F*_ROH_); (c) Total number of runs of homozygosity (ROH) segments per individual; (d) Proportional distribution of ROH lengths across three size classes (0.001–0.5 Mb, 0.5–1 Mb, and 1–3 Mb) for each individual. Pairwise comparisons across populations were tested using the Wilcoxon rank‐sum test with Bonferroni adjusted *p*‐values. Significance levels are indicated as: *p < 0.05, **p < 0.01, ***p < 0.001, ****p < 0.0001, comparisons without asterisks are not statistically significant (*p* ≥ 0.05); (e) Decay of linkage disequilibrium in the five *M. aethiopoides* populations. The plot shows the average *r* ^2^ values between SNP pairs as a function of physical distance (kb).

All populations exhibited similar levels of heterozygosity, with median heterozygosity ranging between ~0.63 and 0.67 (Figure 2a). On the basis of *F*_ROH_ estimates, approximately 5%–10% of the genome consisted of continuous runs of homozygosity across all populations (Figure 2b), with the highest proportion of genome‐wide homozygosity and the greatest number of ROH segments observed in the DUN and MAN populations (Figure 2c), while IRE exhibited the lowest proportion of homozygosity. This aligned with the heterozygosity results, as increased heterozygosity likely breaks up long runs of homozygosity. Finally, 50%–100% of the observed ROH segments across all populations were relatively short (0.01–0.5 Mb) (Figure 2d), indicating that recombination is frequently breaking long homozygous tracts. Notably, the IRE population had fewer ROH and higher heterozygosity compared with the contemporary populations.

LD decay for all populations displayed a rapid initial decline within the first 1 kb, followed by a plateau around *r* ^2^ = 0.22 (Figure 2e), suggesting the presence of recombination, which breaks down LD over time. This pattern was consistent across historic and contemporary populations.

The phylogenetic tree showed three major clusters (Figure S4), consistent with the patterns observed in the PCA. The topology placed individuals within contemporary populations together, with shorter branch lengths—particularly for individuals within the MAN, LIN and DUN populations. The HAM and IRE populations were clearly separated from the rest and showed longer branch lengths.

## 4 Discussion

Our analyses indicated the presence of two distinct New Zealand *M. aethiopoides* genetic clusters, with contemporary populations showing little suggestion of adaptation and diversification following the initial release of the Irish strain. Additionally, genomic patterns of heterozygosity, homozygosity, linkage disequilibrium (LD) and branch length differences in the phylogenetic tree indicates potential facultative sexuality in *M. aethiopoides*, which may have important implications for biocontrol potential in the longer term.

Population structure can arise due to a combination of environmental pressures and intrinsic genetic mechanisms, including variation in reproductive mode (Savolainen et al. 2013; Tvedte et al. 2019). In our study, *M. aethiopoides* exhibited two distinct genetic populations in New Zealand. The Hamilton population clustered closely with the historic Irish population, while the other contemporary populations formed a distinct genetic cluster. This pattern, along with the lack of strong evidence for spatial or temporal divergence in the selection analysis, suggests that environmental factors are not the primary driver of genetic structure. Instead, the observed genetic structure may reflect the distribution and establishment of different released *M. aethiopoides* haplotypes. According to McNeill et al. (2006), eight populations were originally sourced from Ireland, four of which (Crossnacreevy, Athenry, Oakpark and Solohead) were introduced into New Zealand. These comprised two mitochondrial cytochrome oxidase I (COI) haplotypes (Phillips et al. 2006; Vink 2012), which were released across the North and South Islands. Our data suggests that one of these haplotypes may have dispersed more effectively than the other, particularly in the South Island. However, more extensive sampling across both islands is needed to confirm this pattern.

Additive genetic variance (the heritable component of genetic variation that fuels adaptation) is important for the success of biocontrol programmes, as noted by several authors (e.g., Heimpel and Lundgren 2000; Hopper et al. 1993; Stouthamer et al. 1992). Casanovas et al. (2018) further emphasised that additive genetic variance in a biocontrol agent should ideally exceed that of the target host to ensure effective long‐term control. However, in asexual biocontrol agents, such variance is often limited due to low overall genetic diversity, thereby reducing adaptive potential and contributing to inconsistent outcomes compared to their sexual counterparts. Consistent with this, studies on other parasitoids have highlighted that populations with reduced genetic diversity are less capable of adapting to novel environments or evolving host populations (Hopper et al. 2019; Li et al. 2024; Phillips et al. 2008). This is concordant with our findings in *M. aethiopoides*, where low genetic diversity, weak population differentiation, and the absence of spatial or temporal divergence in selection analyses across all regional populations in New Zealand suggest minimal local adaptation. While there is currently no evidence of declining parasitism rates in *S. obsoletus* , the low genetic diversity of the *M. aethiopoides* populations in New Zealand underscores the importance of regular monitoring to detect any sustained reduction in biocontrol effectiveness. Strategically, any early indication of a breakdown in control could prompt targeted efforts to augment genetic diversity through renewed collections of the parasitoid from Ireland.

Genomic data provides a powerful framework for exploring reproductive modes by assessing patterns of homozygosity and heterozygosity across the genome—an approach that has been applied in several other species across multiple studies (e.g., Card et al. 2021; Freitas et al. 2023; Levine and Booth 2025; Mozhaitseva et al. 2023; Sun et al. 2023). Previous studies on the Irish strain of *M. aethiopoides* showed the presence of conserved and actively expressed meiotic genes, along with detectable heterozygosity in the genome, suggesting that this species retains the potential for sexual reproduction (Inwood et al. 2023). In the current study, we observed similarly high heterozygosity in *M. aethiopoides* populations, suggesting that this species mostly reproduces via automixis with central fusion rather than terminal fusion (which would lead to a more rapid loss of heterozygosity) (Alavi et al. 2018; Card et al. 2021; Pearcy et al. 2006). Similar findings have also been observed in the ant *Cataglyphis cursor* (Fonscolombe), where central fusion results in a gradual decline in heterozygosity at rates consistent with theoretical expectations (Pearcy et al. 2006, 2011).

Phylogenetic relationships can reflect the underlying genetic topology of sexual and asexual populations: sexual species with active recombination typically form discrete clades with longer branch separations, while asexual populations more commonly exhibit shorter branches and less discrete structure due to reduced recombination (Tang et al. 2014). In this study, the phylogeny placed MAN, LIN and DUN individuals in a close cluster with shorter branch lengths, whereas HAM and IRE formed separate clusters with longer branch lengths. This pattern likely represents distinct genetic lineages; however, due to the absence of known sexual populations in our dataset, the potential effects of recombination on the branch lengths of the phylogenetic tree cannot be confidently inferred.

Patterns of LD can provide further valuable insights into reproductive modes, as they reflect the extent of recombination and genetic reshuffling (usually driven by sexual reproduction) within a population (Hartfield et al. 2018). In particular, analysing LD decay patterns can help differentiate between obligate and facultative types of asexual reproduction (Freitas et al. 2023; Jaron et al. 2022). For example, in obligate clonal asexual populations, LD typically remains high (*r* ^2^ = 1) due to the complete absence of recombination, whereas in sexual populations, LD decays rapidly (*r* ^2^ < 0.1) (Freitas et al. 2023). The relatively low LD values observed here, together with the high heterozygosity and short homozygous stretches, suggest that *M. aethiopoides* may exhibit facultative sexuality (whereby individuals can alternate between sexual and asexual reproduction depending on host population or environmental cues), similar to that seen in some stick insects (Freitas et al. 2023; Jaron et al. 2022). Such patterns could be expected if LD breaks down rapidly (i.e., high LD decay, as observed here) via recombination (Freitas et al. 2023). Our findings align with the theoretical model by Kuhn et al. (2021), which shows that automictic reproduction, particularly when associated with high heterozygosity and low recombination, can mimic the genetic patterns typically attributed to sexual reproduction. This similarity makes it difficult to detect cryptic sex or transitions between reproductive modes with our current dataset.

To more definitively determine whether sexual reproduction occurs in the Irish strain, it will be necessary to compare both sexual and asexual populations using hybridisation experiments or machine learning‐based genomic models (Kuhn et al. 2021; Levine and Booth 2025; Sun et al. 2023). Such analyses will provide a powerful framework to detect cryptic sex (Freitas et al. 2023; Kuhn et al. 2021; Wachi et al. 2021), characterise the reproductive system and assess its implications for the long‐term effectiveness of *M. aethiopoides* as a biological control agent. Simulation‐based genomic approaches (e.g., using tools like SLiM4; Haller and Messer 2023) could also enable future studies to explore how different parasitoid asexual reproductive modes (such as automixis with central fusion, automixis with terminal fusion, gamete duplication and apomixis), compare with sexual reproduction in shaping long‐term adaptation alongside a sexually reproducing host under coevolutionary pressures. Similar approaches have been used recently to predict species invasiveness (e.g., Camus et al. 2024).

In the context of biological control, the reproductive dynamics of introduced parasitoid populations can be important for determining their long‐term adaptability and effectiveness at both intra‐ and inter‐specific levels. In New Zealand, the sexually reproducing Moroccan strain may be found co‐occurring with the Irish strain of *M. aethiopoides* in pastures (McNeill et al. 2002). If the Irish strain retains the potential for sexual reproduction, as suggested by our data, then occasional gene flow with co‐existing sexual strains could enhance its genetic diversity and potentially improve adaptive capacity (Halkett et al. 2008; Li et al. 2015; Schneider et al. 2003). However, such gene flow also poses risks for biocontrol, as laboratory studies have shown that inter‐ecotype hybridisation can reduce host specificity and undermine the effectiveness of *M. aethiopoides* as a biological control agent (Goldson et al. 2003).

Alongside genetic diversity, gene flow and reproduction, there are other factors that may contribute to biocontrol success. For example, environmental stress, including changes in temperature, can affect key life history traits, such as fecundity, lifespan and development (Amat et al. 2006, 2017; Du et al. 2023; Tenguri et al. 2016; Wang and Smith 1996). In the parasitoid wasp, *Venturi canescens* (Gravenhorst), sexually reproducing (arrhenotokous) females exhibited greater behavioural flexibility under thermal stress, increasing their foraging activity in response to temperature drops, whereas asexual (thelytokous) females showed little behavioural change, suggesting enhanced thermal responsiveness in the sexual strain (Amat et al. 2006). In contrast, a study on the parasitoid wasp, *Diglyphus wani* (Liu, Zhu & Yefremova), found that asexual strains outperformed sexual strains, exhibiting higher fecundity and reproductive rates, and thus potentially greater biocontrol effectiveness under stable environmental conditions (Du et al. 2023). Such studies provide valuable insights into how reproductive mode can influence ecological adaptability, highlighting the role of species‐specific reproductive biology in shaping responses to environmental stresses and informing the effectiveness of biocontrol. Integrating molecular approaches, such as gene expression profiling via PCR and RNA sequencing, into such work could offer deeper insights into the underlying stress responses and adaptive potential of asexual and sexual lineages at the molecular level (Ding et al. 2021; Liu et al. 2020; Song et al. 2020; Xiong et al. 2024).

## Author Contributions

**Meeran Hussain:** conceptualization (equal), data curation (lead), formal analysis (lead), methodology (lead), writing – original draft (lead). **Elahe Parvizi:** conceptualization (equal), formal analysis (supporting), supervision (supporting), writing – review and editing (supporting). **Mark R. McNeill:** conceptualization (supporting), data curation (supporting), writing – review and editing (supporting). **Ang McGaughran:** conceptualization (equal), formal analysis (supporting), funding acquisition (lead), resources (lead), supervision (lead), writing – review and editing (supporting).

## Conflicts of Interest

The authors declare no conflicts of interest.

## Supporting information


**Appendix S1:** ece372533‐sup‐0001‐AppendixS1.docx.

## Data Availability

Raw demultiplexed whole‐genome resequencing reads are available in the NCBI Sequence Read Archive (BioProject no.: PRJNA1260570). All analysis scripts and metadata file used in this study are available at GitHub: https://github.com/meeranhussain/Population_genomic_analysis.
