# Supplementary material for: Using Genomic Data to Infer Evolutionary Processes in the Asexual Parasitoid Microctonus aethiopoides
Source: Ecol Evol. 2025 Dec 26;15(12):e72533. doi: 10.1002/ece3.72533 (PMC12742435; doi:10.1002/ece3.72533)
Supplement: Supplementary file 1 — Appendix S1: ece372533‐sup‐0001‐AppendixS1.docx. [file ECE3-15-e72533-s001.docx]

Using genomic data to infer evolutionary processes in the asexual parasitoid *Microctonus aethiopoides*

Meeran Hussain^1^, Elahe Parvizi^1^, Mark R. McNeill^2^, Ang McGaughran^1^*

^1^Te Aka Mātuatua - School of Science, University of Waikato, Private Bag 3105, Hamilton, 3240, New Zealand

^2^Bioeconomy Science Institute, Tuhiraki, Christchurch, New Zealand

**Corresponding author:* amcgaugh@waikato.ac.nz

Meeran Hussain: meeranhussain1996@gmail.com

Elahe Parvizi: ellie.parvizi@waikato.ac.nz

Mark R. McNeill: mark.mcneill@agresearch.co.nz

Ang McGaughran: amcgaugh@waikato.ac.nz

**Table S1.** Details of all samples used in this study, including collection sites, sample type, and DNA concentration (ng) measured prior to sequencing

| **Sl. no** | **Location** | **Latitude** | **Longitude** | **Sample type** | **Collection date** | **Sample-ID** | **Eluted volume (ul)** | **Qubit (ng/ul)** | **Qubit Conc (ng)** |
| --- | --- | --- | --- | --- | --- | --- | --- | --- | --- |
| 1 | Mangōnui | -35.01041445 | 173.5992881 | Larvae | 16/05/2024 | MAN-01 | 55 | 8 | 440 |
| 2 | Mangōnui | -35.01041445 | 173.5992881 | Larvae | 16/05/2024 | MAN-02 | 55 | 5.1 | 280.5 |
| 3 | Mangōnui | -35.01041445 | 173.5992881 | Larvae | 16/05/2024 | MAN-03 | 55 | 14.8 | 814 |
| 4 | Mangōnui | -35.01041445 | 173.5992881 | Larvae | 16/05/2024 | MAN-04 | 55 | 10.2 | 561 |
| 5 | Mangōnui | -35.01041445 | 173.5992881 | Larvae | 16/05/2024 | MAN-05 | 55 | 16.7 | 918.5 |
| 6 | Hamilton | -37.77696193 | 175.3126127 | Larvae | 11/03/2024 | HAM-01 | 55 | 5.68 | 312.4 |
| 7 | Hamilton | -37.77696193 | 175.3126127 | Larvae | 11/03/2024 | HAM-02 | 55 | 6.44 | 354.2 |
| 8 | Hamilton | -37.77696193 | 175.3126127 | Larvae | 11/03/2024 | HAM-03 | 55 | 7.88 | 433.4 |
| 9 | Hamilton | -37.77696193 | 175.3126127 | Larvae | 11/03/2024 | HAM-04 | 55 | 9.86 | 542.3 |
| 10 | Hamilton | -37.77696193 | 175.3126127 | Larvae | 11/03/2024 | HAM-05 | 55 | 8.86 | 487.3 |
| 11 | Hamilton | -37.77696193 | 175.3126127 | Larvae | 11/03/2024 | HAM-06 | 55 | 5.28 | 290.4 |
| 12 | Hamilton | -37.77696193 | 175.3126127 | Larvae | 11/03/2024 | HAM-07 | 55 | 8.16 | 448.8 |
| 13 | Hamilton | -37.77696193 | 175.3126127 | Larvae | 11/03/2024 | HAM-08 | 55 | 11.5 | 632.5 |
| 14 | Hamilton | -37.77696193 | 175.3126127 | Larvae | 11/03/2024 | HAM-09 | 55 | 6.86 | 377.3 |
| 15 | Hamilton | -37.77696193 | 175.3126127 | Larvae | 11/03/2024 | HAM-10 | 55 | 5.38 | 295.9 |
| 16 | Lincoln | -43.64262001 | 172.4689808 | Larvae | 23/08/2023 | LIN-01 | 55 | 15 | 825 |
| 17 | Lincoln | -43.64262001 | 172.4689808 | Larvae | 23/08/2023 | LIN-02 | 55 | 18 | 990 |
| 18 | Lincoln | -43.64262001 | 172.4689808 | Larvae | 23/08/2023 | LIN-03 | 55 | 8.8 | 484 |
| 19 | Lincoln | -43.64262001 | 172.4689808 | Larvae | 23/08/2023 | LIN-04 | 55 | 24 | 1320 |
| 20 | Lincoln | -43.64262001 | 172.4689808 | Larvae | 23/08/2023 | LIN-05 | 55 | 17 | 935 |
| 21 | Lincoln | -43.64262001 | 172.4689808 | Larvae | 23/08/2023 | LIN-06 | 55 | 9 | 495 |
| 22 | Lincoln | -43.64262001 | 172.4689808 | Larvae | 23/08/2023 | LIN-07 | 55 | 8.56 | 470.8 |
| 23 | Lincoln | -43.64262001 | 172.4689808 | Adult | 23/08/2023 | LIN-08 | 55 | 3.56 | 195.8 |
| 24 | Lincoln | -43.64262001 | 172.4689808 | Adult | 23/08/2023 | LIN-09 | 55 | 2.34 | 128.7 |
| 25 | Lincoln | -43.64262001 | 172.4689808 | Adult | 23/08/2023 | LIN-10 | 55 | 4.26 | 234.3 |
| 26 | Dunedin | -45.85701779 | 170.3915749 | Larvae | 07/09/2023 | DUN_01 | 55 | 9.22 | 507.1 |
| 27 | Dunedin | -45.85701779 | 170.3915749 | Larvae | 07/09/2023 | DUN_02 | 55 | 5.78 | 317.9 |
| 28 | Dunedin | -45.85701779 | 170.3915749 | Larvae | 07/09/2023 | DUN_03 | 55 | 13.7 | 753.5 |
| 29 | Dunedin | -45.85701779 | 170.3915749 | Larvae | 07/09/2023 | DUN_04 | 55 | 10.3 | 566.5 |
| 30 | Dunedin | -45.85701779 | 170.3915749 | Larvae | 07/09/2023 | DUN_05 | 55 | 4.26 | 234.3 |
| 31 | Dunedin | -45.85701779 | 170.3915749 | Larvae | 07/09/2023 | DUN_06 | 55 | 13.6 | 748 |
| 32 | Dunedin | -45.85701779 | 170.3915749 | Larvae | 07/09/2023 | DUN_07 | 55 | 13.3 | 731.5 |
| 33 | Dunedin | -45.85701779 | 170.3915749 | Larvae | 07/09/2023 | DUN_08 | 55 | 7.3 | 401.5 |
| 34 | Dunedin | -45.85701779 | 170.3915749 | Larvae | 07/09/2023 | DUN_09 | 55 | 6.4 | 352 |
| 35 | Dunedin | -45.85701779 | 170.3915749 | Larvae | 07/09/2023 | DUN_10 | 55 | 9 | 495 |
| 36 | Ireland (Athenry) | 53.30119371 | -8.746320598 | Adult | 2002 | IRE-01 | 55 | 3.16 | 173.8 |
| 37 | Ireland (Athenry) | 53.30119371 | -8.746320598 | Adult | 2002 | IRE-02 | 55 | 3.06 | 168.3 |
| 38 | Ireland (Athenry) | 53.30119371 | -8.746320598 | Adult | 2002 | IRE-03 | 55 | 3.04 | 167.2 |
| 39 | Ireland (Athenry) | 53.30119371 | -8.746320598 | Adult | 2002 | IRE-04 | 55 | 3.02 | 166.1 |
| 40 | Ireland (Athenry) | 53.30119371 | -8.746320598 | Adult | 2002 | IRE-05 | 55 | 2.62 | 144.1 |
| 41 | Ireland (Athenry) | 53.30119371 | -8.746320598 | Adult | 2004 | IRE-06 | 55 | 2.06 | 113.3 |
| 42 | Ireland (Athenry) | 53.30119371 | -8.746320598 | Adult | 2004 | IRE-07 | 55 | 2.78 | 152.9 |
| 43 | Ireland (Athenry) | 53.30119371 | -8.746320598 | Adult | 2004 | IRE-08 | 55 | 2.54 | 139.7 |
| 44 | Ireland (Athenry) | 53.30119371 | -8.746320598 | Adult | 2001 | IRE-09 | 60 | 2.5 | 150 |

**Table S2.** Summary of read mapping statistics for each sample of *Microctonus aethiopoides*. The table includes the sample ID, the total number of reads retained after quality filtering, the number of reads mapped to the reference genome, and the corresponding percentage of mapped reads. MAN = Mangonui, HAM =Hamilton, LIN = Lincoln, DUN = Dunedin, IRE = Ireland.

| **Sl.no** | **Sample** | **Total reads after filtration (150*2)** | **No reads mapped (150*2)** | **Mapping (%)** |
| --- | --- | --- | --- | --- |
| 1 | MAN_01 | 36,453,952 | 36,101,707 | 99.0 |
| 2 | MAN_02 | 34,251,174 | 33,977,958 | 99.2 |
| 3 | MAN_03 | 35,171,518 | 34,928,188 | 99.3 |
| 4 | MAN_04 | 23,266,006 | 22,968,128 | 98.7 |
| 5 | MAN_05 | 47,288,376 | 46,716,325 | 98.8 |
| 6 | HAM_01 | 55,992,644 | 54,849,757 | 98.0 |
| 7 | HAM_02 | 24,800,414 | 24,234,959 | 97.7 |
| 8 | HAM_03 | 20,087,704 | 19,940,912 | 99.3 |
| 9 | HAM_04 | 41,273,268 | 40,337,496 | 97.7 |
| 10 | HAM_05 | 24,639,488 | 24,452,818 | 99.2 |
| 11 | HAM_06 | 21,372,098 | 20,013,901 | 93.6 |
| 12 | HAM_07 | 36,391,030 | 35,932,084 | 98.7 |
| 13 | HAM_08 | 43,846,808 | 43,225,397 | 98.6 |
| 14 | HAM_09 | 29,684,160 | 29,107,958 | 98.1 |
| 15 | HAM_10 | 38,648,384 | 38,103,580 | 98.6 |
| 16 | LIN_01 | 26,368,138 | 25,691,336 | 97.4 |
| 17 | LIN_02 | 35,206,002 | 34,537,238 | 98.1 |
| 18 | LIN_03 | 29,002,534 | 28,687,208 | 98.9 |
| 19 | LIN_04 | 25,173,044 | 24,887,899 | 98.9 |
| 20 | LIN_05 | 40,098,562 | 39,356,289 | 98.2 |
| 21 | LIN_06 | 24,721,446 | 24,403,279 | 98.7 |
| 22 | LIN_07 | 37,718,142 | 37,084,014 | 98.3 |
| 23 | LIN_08 | 30,028,108 | 26,782,060 | 89.2 |
| 24 | LIN_09 | 25,525,434 | 20,234,417 | 79.3 |
| 25 | LIN_10 | 51,014,722 | 44,782,596 | 87.8 |
| 26 | DUN_01 | 26,313,144 | 25,854,306 | 98.3 |
| 27 | DUN_02 | 40,416,140 | 39,254,919 | 97.1 |
| 28 | DUN_03 | 26,345,776 | 25,130,862 | 95.4 |
| 29 | DUN_04 | 74,662,184 | 73,055,045 | 97.9 |
| 30 | DUN_05 | 34,370,342 | 33,627,857 | 97.8 |
| 31 | DUN_06 | 34,445,638 | 33,646,636 | 97.7 |
| 32 | DUN_07 | 39,667,840 | 38,818,325 | 97.9 |
| 33 | DUN_08 | 32,874,472 | 32,591,054 | 99.1 |
| 34 | DUN_09 | 25,984,624 | 25,479,018 | 98.1 |
| 35 | DUN_10 | 25,158,904 | 24,721,560 | 98.3 |
| 36 | IRE_01 | 30,344,284 | 30,140,784 | 99.3 |
| 37 | IRE_02 | 20,157,824 | 20,007,931 | 99.3 |
| 38 | IRE_04 | 46,919,472 | 46,587,448 | 99.3 |
| 39 | IRE_05 | 31,800,998 | 31,577,560 | 99.3 |
| 40 | IRE_06 | 52,465,230 | 52,096,465 | 99.3 |
| 41 | IRE_07 | 16,769,832 | 16,654,113 | 99.3 |
| 42 | IRE_08 | 33,220,002 | 32,988,126 | 99.3 |
| 43 | IRE_09 | 18,304,984 | 18,179,108 | 99.3 |

**Figure S1.** Cross-entropy validation for inference of the optimal number of ancestral populations of *Microctonus aethiopoides* (K).


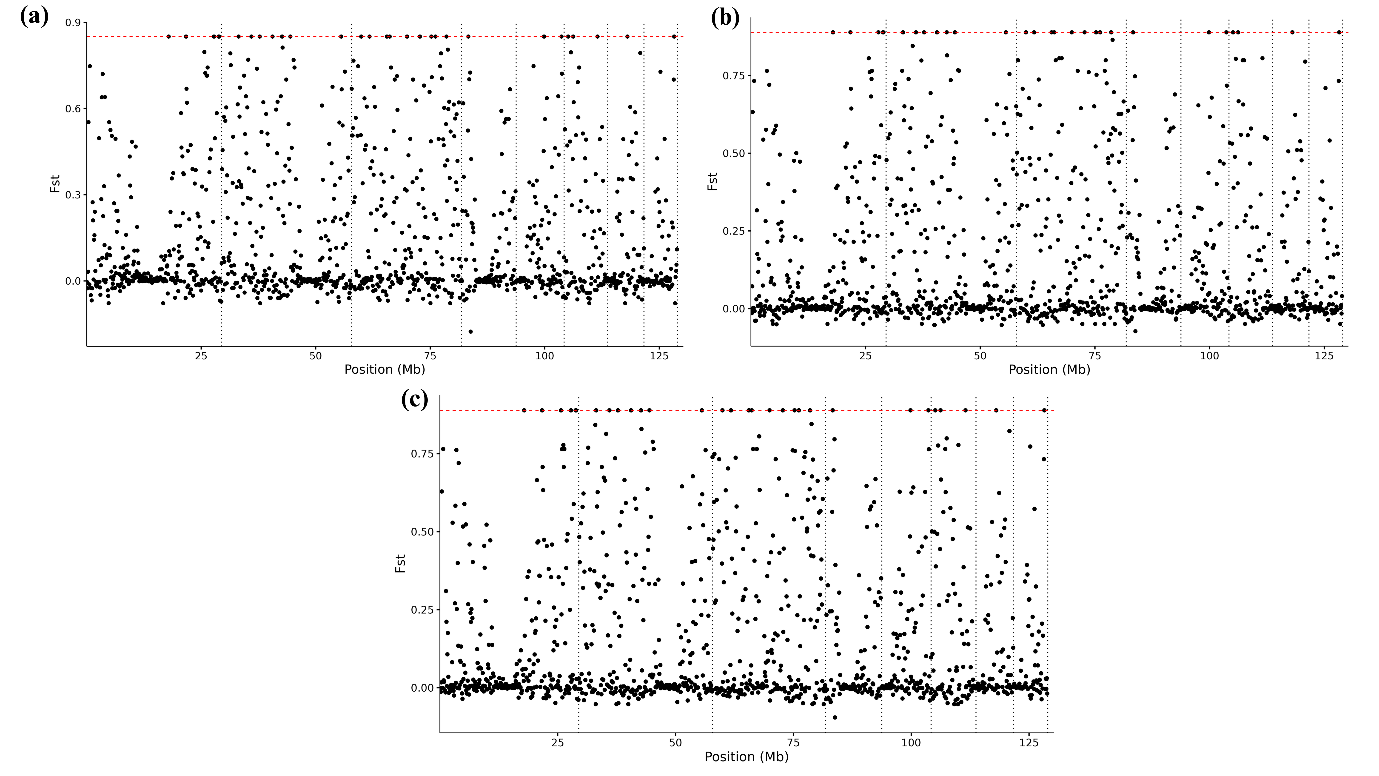


**Figure S2.** Genome-wide distribution of pairwise F_ST_ values for comparisons involving the Hamilton (HAM) population of *Microctonus aethiopoides*. Each panel represents a sliding window analysis of F_ST_ across the genome for the following comparisons: Hamilton vs. Mangonui **(a)**, Hamilton vs. Lincoln **(b)**, and Hamilton vs. Dunedin **(c)**. The red horizontal dotted line indicates the 99th percentile threshold of F_ST_ values, representing the top 1% outliers, and the vertical dotted line represents scaffold boundaries.


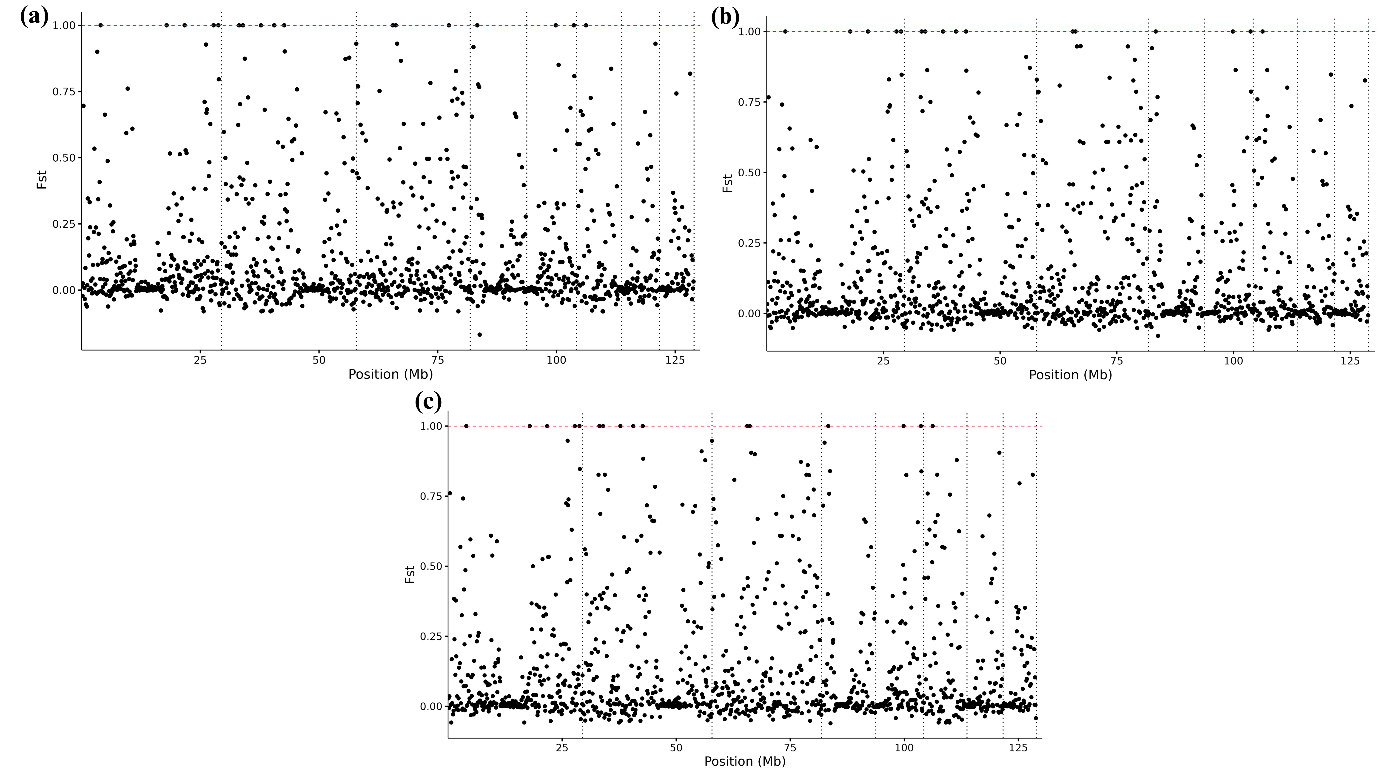


**Figure S3.** Genome-wide distribution of pairwise F_ST_ values for comparisons involving the historic Ireland (IRE) *Microctonus aethiopoides* population compared to the released New Zealand populations. Each panel represents a sliding window analysis of F_ST_ across the genome for the following comparisons: Ireland vs. Mangonui **(a)**, Ireland vs. Lincoln **(b)**, and Ireland vs. Dunedin **(c)**. The red horizontal dotted line indicates the 99th percentile threshold of F_ST_ values, representing the top 1% outliers, and the vertical dotted line represents scaffold boundaries.


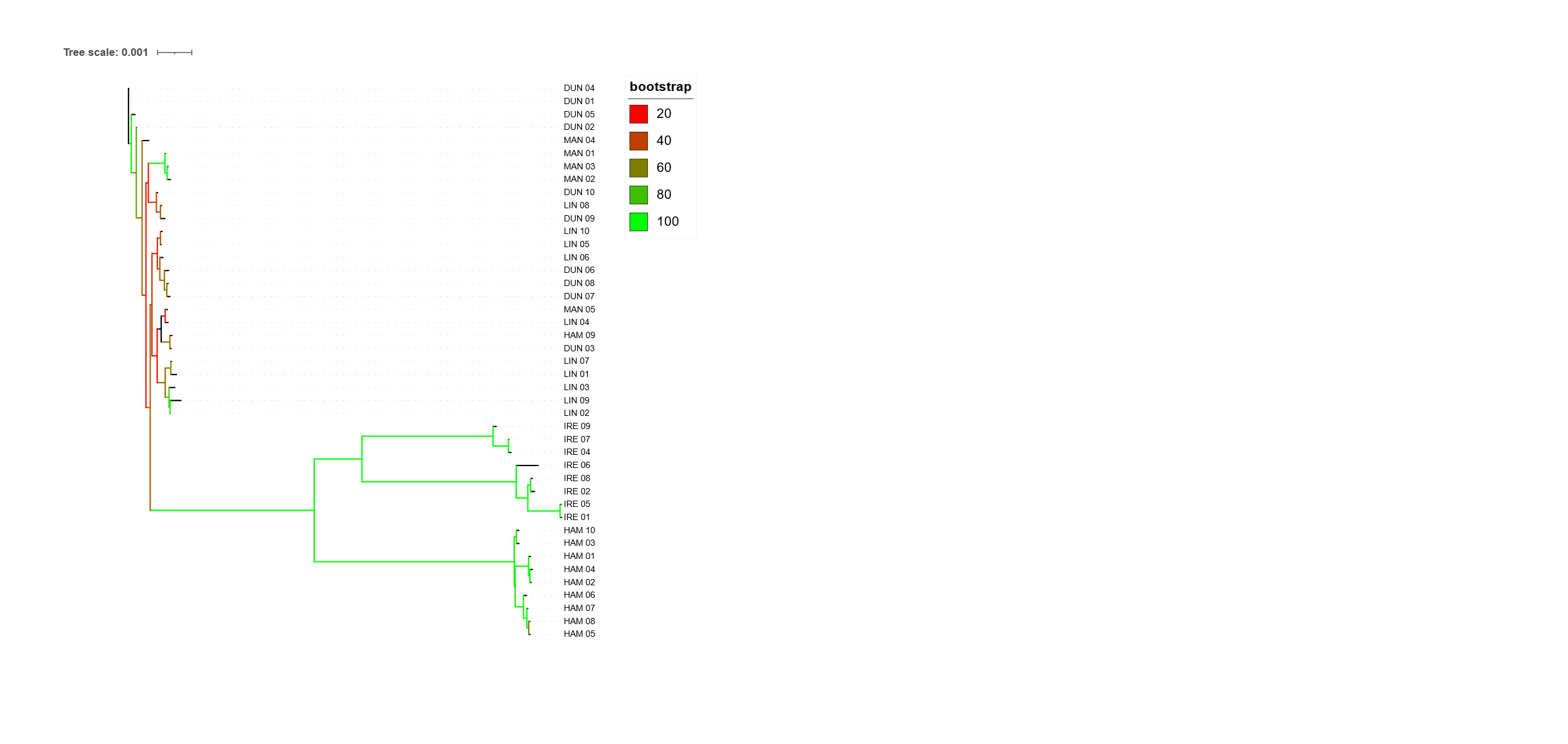


**Figure S4.** Maximum-likelihood phylogenetic tree of *Microctonus aethiopoides* populations from Ireland and New Zealand, based on genome-wide SNP data. Bootstrap support values are indicated by branch colours, ranging from red (low support) to green (high support), as indicated by the provided key.
